# Create Fat Substitute From Soybean Protein Isolate/Konjac Glucomannan: The Impact of the Protein and Polysaccharide Concentrations Formulations

**DOI:** 10.3389/fnut.2022.843832

**Published:** 2022-03-09

**Authors:** Lu Huang, Yuqing Ren, He Li, Qibo Zhang, Yong Wang, Jinnuo Cao, Xinqi Liu

**Affiliations:** ^1^National Soybean Processing Industry Technology Innovation Center, School of Food and Health, Beijing Technology and Business University, Beijing, China; ^2^School of Chemical Engineering, UNSW Sydney, Sydney, NSW, Australia; ^3^Plant Meat (Hangzhou) Health Technology Limited Company, Hangzhou, China

**Keywords:** sensory evaluation, fat substitute, texture, oral tribology, chromaticity

## Abstract

In this study, soybean protein isolate (SPI) and coconut oil were emulsified and konjac flour was added to prepare the protein/polysaccharide composite emulsion gel. The SPI/polysaccharide compound fat substitute was obtained by vacuuming. The effects of protein and konjac flour addition on the gel system of the mixed emulsion were explored. Sensory evaluation experiments showed that the overall acceptability of fat substitutes added with 1% SPI was higher. With the increase of protein and konjac content, the juiciness of the samples decreased gradually. The increase of konjac content reduced the brightness of compound fat substitutes, and the yellowness of compound fat substitute increases significantly with the increase of protein content. The rheological results showed that the G′ and loss modulus (G″) increased with the increase of protein and konjac content, forming a rigid elastic gel matrix, which provided a basis for the preparation of fat substitutes. Texture profile analysis (TPA) results showed that the springiness of all samples was similar to the natural fat after 20 min of heating. With the increase of protein and konjac content, the hardness of the samples increased gradually. The results of oral tribology showed that the friction coefficients of all samples were very small. The friction behavior of the samples with SPI content of 1% was similar to that of natural fat, which could better simulate the swallowing feeling and lubricity of natural fat. To sum up, the appearance of solid fat substitutes prepared with SPI and konjac flour is similar to pork fat. They show ideal functional characteristics in mechanical properties and oral tribology. Among them, the fat substitute with the protein content of 1% and konjac content of 4% is the most popular among consumers.

## Introduction

Traditional animal meat production is struggling to meet the demand of an increasing global population. According to Dupont et al. the world's population is expected to increase to 11.2 billion by the year 2100, which will lead to increased meat consumption, especially in developing countries (1). Therefore, to meet the protein production of the emerging population, the environmental challenges caused by animal meat demand are on the rise, while the negative impact of meat products on health is driving the rapid expansion of the plant-based meat market (2). Evidence shows that excessive intake of animal fat, especially the red meat from pork and beef, can cause chronic diseases, obesity, and cardiovascular disease (3). Although the pathogenesis of the cardiovascular disease is complex and involves many factors, dietary intervention seems to be one of the most popular and simplest preventative strategies (4). The main component of pork fat is saturated fatty acids, which has a negative impact on healthy diet of human (5). Consequently, plant-based diet is attracting more attention from consumers, researchers and decision-makers because they have the potential to prompt healthy diet, protect the environment and animal welfare (6). Furthermore, with an increasing focus on a healthy diet and body management in recent years, consumers have been demanding healthy low-fat products as they make better lifestyle choices (7). Fat substitutes can mimic the taste and texture of fat without potential harm to health due to the high fat content, so they have attracted a lot of research interest (8).

Soybean is a high-quality plant protein resource (9). The SPI has excellent functionalities such as gelation, emulsification and it can also accelerate the metabolism of fat and energy to prevent obesity and reduce cholesterol (10). SPI has also been used to produce emulsion gels with excellent freeze-thaw stability and rheological properties, implying the potential to mimic animal fat tissues (11). Mixing SPI and polysaccharide could improve the emulsification capabilities (12). In particular, it has been reported that SPI mixed with cellulose nanofiber can provide a texture and mouthfeel similar to animal fat as shown in a previous study (13). Konjac glucomannan is a neutral polysaccharide extracted from konjac, a native plant in East Asia. It has significant benefits to human health as dietary fiber, and can be beneficial for weight control (14). It also has the functions of preventing hypercholesterolemia, anti-inflammatory and prebiotic activities (15). It has been reported that gel formed by konjac glucomannan combined with carrageenan, can be used as a “fat analog” in low-fat meat products (16). Transglutaminase (TG) has the function of stabilizer, emulsifier and thickener, and avoids the adverse effects of gel products, such as dehydration, so it is often used as food additive (17).

Many studies have explored various fat reduction strategies. Barbut et al. used different organic ethylcellulose vegetable oil gels to replace porcine liver fat, which showed similar sensory characteristics as animal fat and less odor while successfully reducing the saturated fat content by 62% (18). Zetzl et al. modified the ethyl cellulose gels, which not only retain solid structure in frankfurter sausage without significant difference in chewing and hardness, but also has a healthier fatty acid composition than the control product made of beef fat (19). Gravelle et al. captured liquid oil in gel networks and used these structures to simulate the properties of solid animal fat. It contains more unsaturated fatty acids and improves the nutritional status of the product (20). Serdaroglu used olive oil, gelatin and inulin as raw materials to obtain an emulsion gel. The addition of 50% emulsion has similar cooking characteristics to the fat-free beef fat substitutes, and the emulsion gel significantly affects the color parameters and texture behavior of the samples (21). Leng et al. used SPI and sunflower oil emulsion to replace pig back fat to in frankfurter sausages, and was able to decrease the energy of frankfurters from 1,027.86 to 816.16 kJ/100 g (22). Francisco et al. prepared dry fermented sausage using a healthier oil mixture (olive oil, flax oil, and fish oil) stabilized in a konjac matrix to replace pig back fat. Consequently, the saturated fatty acid content and hardness were decreased, while the polyunsaturated fatty acid and adhesion increased without compromising the springiness and chewability (23).

Fat substitute prepared using different proteins or polysaccharides have reportedly been added to various meat products, including sausages and patties. However, few studies have involved simulating pork fat products with optimized three-dimensional structures. To the best of our knowledge, there is no report regarding the oral tribology of protein/polysaccharide composite fat substitute. Therefore, the current study aims to develop an SPI/polysaccharide compound fat substitute that can better mimic natural pork fat in appearance and taste, without compromising mechanical, rheological, and thermal properties. Particularly the impact of the konjac and SPI content on the optimal quality will be evaluated in details by instruments and sensory experiment.

## Materials and Methods

### Materials

The SPI was purchased from the Shandong Yuwang Plant Protein Co., Ltd. (Shandong, China), with protein content of >91% on a dry basis according to the manufacturer. And the coconut oil was obtained from the Henan Yuanzhuo Biotechnology Co., Ltd. (Henan, China). The konjac powder was provided by the Hubei Consistent Biotechnology Co., Ltd. (Hubei, China), with glucomannan content on a dry basis of >80% according to the manufacturer. The cassava starch was purchased from the Shandong Zhanze Biotechnology Co., Ltd. (Shandong, China), and the transglutaminase (TG) enzymes were acquired from the Guangdong Kelong Biotechnology Co., Ltd. (Guangdong, China). Pork back fat was obtained from a local market (Beijing, China) and stored at 4°C in fridge before use.

### Preparation of the Protein/Polysaccharide Emulsion Gel

The SPI powder was evenly dispersed in distilled water (0–5°C) and stirred well-using a magnetic stirrer for 5 min. An electronic stirrer (EUROSTAR 40 digital, IKA, Baden-Württemberg, Germany) was then used to stir the mixture for 5 min at 6,000 rpm, after which coconut oil was added at 9,000 rpm for 7 min to emulsify. Konjac flour was then gradually added and stirred at 3,000 rpm for 5 min. The cassava starch and TG were dissolved separately in water and blended thoroughly at 4,000 rpm for 3 min using an electronic mixer. The mixture was incubated for 30 min at 50°C in a water bath, inducing protein cross-linking gelation to obtain the protein/polysaccharide emulsion gel.

### Preparation of the Fat Substitutes

After incubation, the samples were poured into a diagonal 5-inch baking mold, degassed at −0.09 MPa for 30 s (exelway, DZ-300, Quanzhou Liding mechanical equipment Co., Ltd, Fujian, China), cooked at 95°C for 60 min (Jintan Kexi, HH-2 Water bath pot, Jintan Kexi Instrument Co., Ltd, Jiangsu, China), and then stored for 24 h in a refrigerator at 4°C for further analysis.

### The Morphology and Photographic Images of Fat Substitutes

The prepared samples were cut into small pieces of 1 cm × 1 cm × 1 cm and pre-frozen in a refrigerator at −40°C for 24 h. The pieces were then placed in a vacuum freeze-dryer (Marin Christ, Beta 1–8 LSC basic, Christ, Osterode, Germany) for 48 h until fully dried and ground into small particles using a planetary mill (JX-4G, Shanghai Jingxin Technology Co., Ltd, Shanghai, China). The samples were attached to a double-sided conductive copper sample holder with adhesive tape and sprayed with gold (ETD-2,000c ion sputtering evaporation instrument, Beijing Boyuan Weina Technology Co., Ltd, Beijing, China). Scanning electron microscopy (Zeiss Gemini 300 SEM, Carl Zeiss, Jena, Germany) was then used for observation at 500x and 1,000x magnification (the accelerating voltage is 1.5 kV and the signal source is secondary electron), while representative visual fields were selected for imaging. The prepared samples were cut into small pieces of 3 cm × 3 cm × 1 cm, and photographed for visual assessment.

### Sensory Evaluation of the Fat Substitutes

The sensory evaluation experiment was conducted by 15 untrained consumers (10 women and 5 men; aged from 18 to 40) in a constant temperature (26°C) sensory evaluation room. The samples were stored at 4°C and equilibrated at room temperature (26°C) for 1 h before the sensory test. S1K2 represents the sample of fat substitute containing SPI 1% and konjac 2%, S1K4 represents the sample of fat substitute containing SPI 1% and konjac 4% and so on. The samples represented by each abbreviation are shown in Table 1. The team members were asked to evaluate the juiciness, chewiness, texture status and overall acceptability of the samples on a 10-point scale, ranging from very ideal (10 points) to unsatisfactory (1 point) (24), and the team members were asked to give different scores to six samples. After every sample tasting, the evaluators were required to rinse their mouths with water to prevent experimental errors caused by taste fatigue.

**Table 1:** Each name represents the raw material composition of the sample.

**Sample name**	**Coconut oil (%)**	**SPI (%)**	**Konjac glucomannan (%)**	**Starch (%)**	**TG (%)**
S1K2	10	1	2	12	1.5
S1K4	10	1	4	12	1.5
S1K6	10	1	6	12	1.5
S5K2	10	5	2	12	1.5
S5K4	10	5	4	12	1.5
S5K6	10	5	6	12	1.5

### Texture Analysis of the Fat Substitutes

The method described by Dreher et al. was slightly modified to determine the mechanical properties of fat substitutes (25). The samples were cut into 3 cm × 3 cm × 3 cm cubes for texture profile analysis (TPA) using a texture analyzer (CT3, Brookfield, Middleboro, USA). The samples were compressed axially using a TA10 probe (12.7 mm in diameter and 35 mm in length), while 5 s was allowed between two compression cycles. Uniaxial compression was performed twice at a test speed of 1 mm/s to deform the samples to 40% of its original height at a trigger point load of 5 g. Record the change of load with time with the instrument's own software TexturePro CT to obtain the hardness, cohesiveness and springiness values. Each sample was evaluated ten times.

### Oral Tribology of the Fat Substitutes

The samples were cut into small pieces and stirred for 1 min at 5,000 rpm in a cooking machine (Vorwerk, Meishanpin multifunctional food processor TM5, Vorwerk, Wuppertal, Germany) to obtain the fat substitute particles. Then, 3 mL of artificial saliva (Source leaf, ISO/TR10271, neutral, Shanghai Yuanye Biotechnology Co., Ltd, Shanghai, China) was added to 3 gram of samples to simulate the food state during swallowing. The friction curve was measured using a friction fixture with a stress-controlled rheometer (DHR-1, TA instruments, New Castle, USA). The rough hydrophobic surface of 3M Transpore Surgical Tape 1527–2 was considered as the state with a comparable surface roughness and wettability to the human tongue (26). A 3M™ Transpore™ Surgical Tape, 1527-2, 50 mm ×9.1 m, (3M Medical, USA) was used to simulate the tongue. The tape was cut in a square form, located, and compacted tightly on top of the lower plate rheometry. After each experiment, the tape was changed and the rheometer was cleaned and dried with deionized water and laboratory wipes. The normal force exerted on the samples during oral processing was simulated with using 2N axial force. Before samples loading, the tape was cut into a square and pasted onto the flat base, while the surplus part was attached to the exterior of the base. The tape was replaced, and the base was cleaned after each measurement. Test conditions: Temperature 37°C, stress 2N (pressure 27.83 kPa), and speed 0.01 s^−1^ to 100 s^−1^. The equilibration time on the machine was set the same for each sample (5 s), while the measurements were repeated three times.

### Color Measurement of the Fat Substitutes

The samples and natural pork fat were cut into 3 cm ×3 cm ×1 cm chunks, after which a CM-3610A colorimeter (Konica Minolta, Japan) was used to analyze their color. The instrument shall be calibrated with a white board (standard reference provided by Konica Minolta) and a standard black board before use. The detection light source is D65 pulsed xenon lamps. The objective color CIE-LAB tristimulus values, L^*^ (lightness), a^*^ (red/green axis), and b^*^ (yellow/blue axis) parameters were recorded. Each sample was subjected to 10 parallel determinations.

### Differential Scanning Calorimetry Analysis

A differential scanning calorimeter (DSC-60 Plus, Shimadzu, Japan) was used to analyze the thermal behaviors of the samples in the temperatures range from 40°C to 200°C. About 10 mg of the samples was placed into a liquid crucible and sealed by DSC-60A seal kit (Shimadzu, Japan). The initial temperature of the samples was 40°C, after which it was heated to 200°C at a heating rate of 10°C/min.

### Rheological Analysis

The rheological behavior of the samples was determined using stress-controlled rheometer (DHR-1, TA Instruments, New Castle, US) equipped with a Peltier temperature control device. The rheological properties of the protein/polysaccharide composite emulsion gel were measured following the method described by Gao et al. (27). The surface of the measuring system was sandblasted to avoid wall sliding, while the gap was set to 1 mm, and the measurement temperature was set to 37°C for all measurement. About 4 g sample was collected from the center of each sample and placed in the middle of the plate. The top plate was gradually lowered until it was 1 mm from the bottom plate. The redundant sample portion was removed using a scraper, after which the sample were covered by a thin layer methyl silicone oil to the edges to prevent evaporation. After stirring, the gel was subjected to temperature scanning. Temperature scanning conditions: Frequency 10 rad/s^−1^, strain 1%, temperature range 50–100°C, heating rate 5°C/min. The linear viscoelastic region was determined by amplitude sweep over the strain range of 0.01–60% (data not shown). The gel was then subjected to frequency scanning (angular frequency range 6.28–62.8 rad/s) to determine the rheological behavior of the samples in the linear viscoelastic (LVE) region.

### Data Analysis

Origin 2021 software was used for data processing, and SPSS software was used for one-way ANOVA. *P* <0.05 was considered as significantly different, and all measurements were repeated for at least three times.

## Results and Discussion

### Morphology and Photographic Image

**Figure 1:**
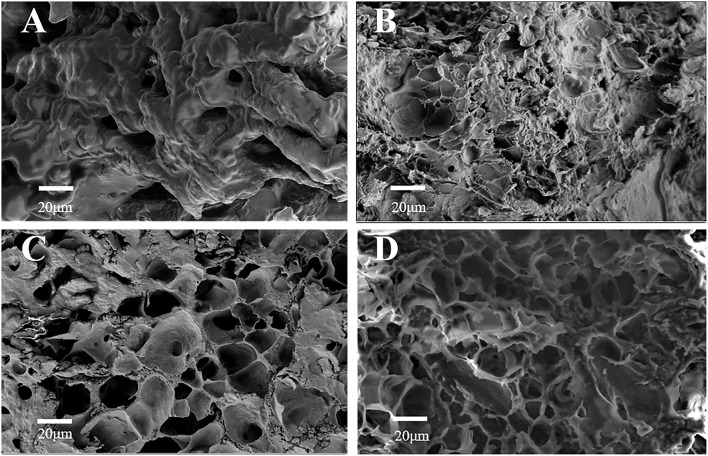
SEM observation of fat substitutes with different formulations, whereas **(A)** SPI/coconut oil emulsion (S5K4). **(B)** The addition of konjac. **(C)** The addition of starch. **(D)** The addition of TG (S5K4).

Figure 1 shows the SEM result of each material added to S5K4. Figure 1A shows the microstructure of SPI/coconut oil emulsion. The relatively high SPI concentration (5%) caused the formation of a protein network gel. Figure 1B shows the microstructure of the sample with Konjac flour. The protein network is partially filled with konjac, displaying a solid matrix with a few holes on the surface. Figure 1C illustrates the sample microstructure after adding cassava star, revealing many meshes in the sample matrix, which may be caused by starch gelatinization. Figure 1D shows the sample microstructure after adding TG, displaying a porous sample surface and an uneven honeycomb morphology with small holes inside. These holes were connected to form a dense network structure. This is because transglutaminase catalyzes glutamine residues in the protein side chain γ- Hydroxylamine group and lysine ε-Covalent cross-linking between amino groups, so a large number of dense three-dimensional network structures are formed (28). Its structure primarily consisted of a dense network formed by konjac, supplemented by the SPI network structure and the three-dimensional starch network structure.

**Figure 2:**
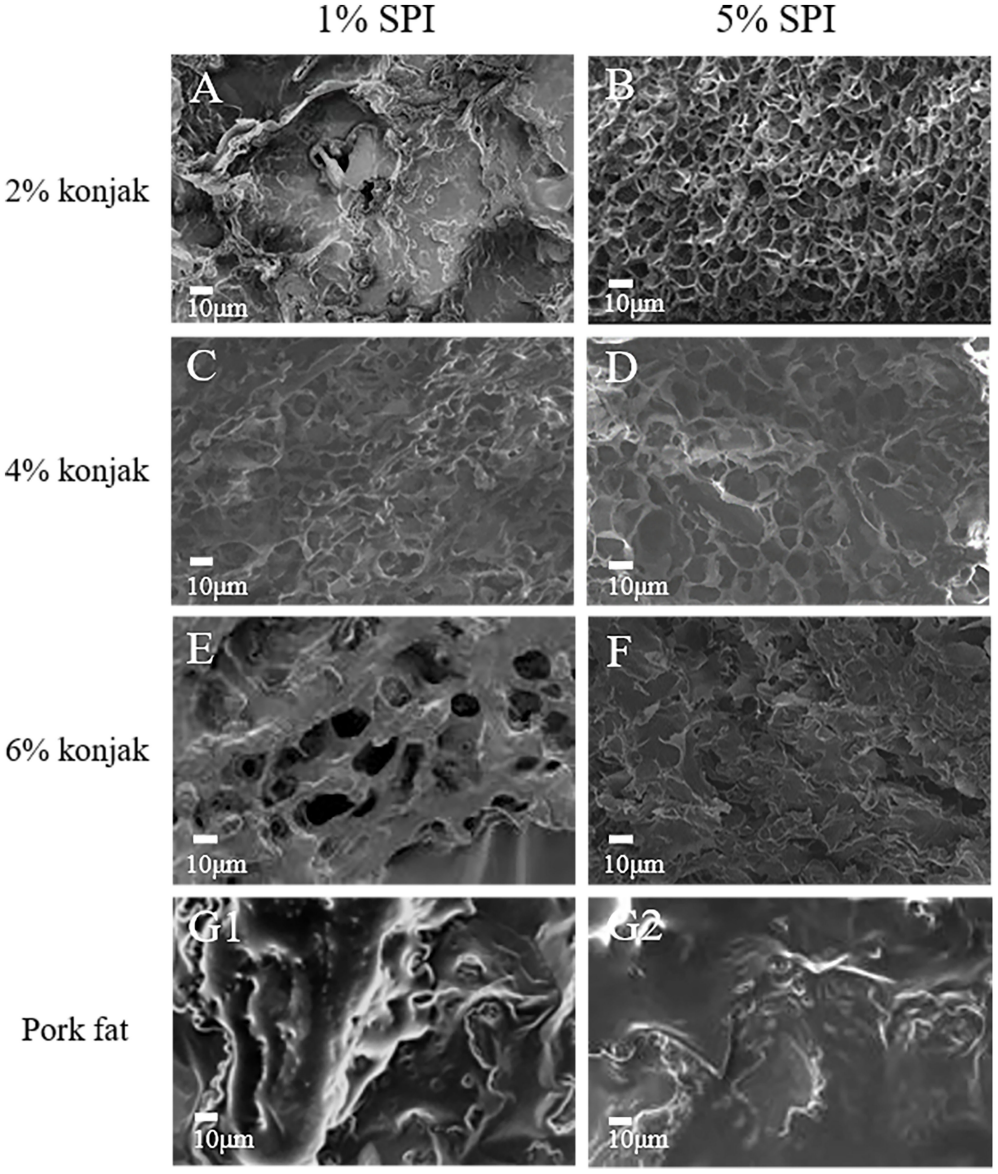
The microstructures of samples with different protein content and konjac content. **(A)** S1K2; **(B)** S5K2; **(C)** S2K4; **(D)** S5K4; **(E)** S1K6; **(F)** S5K6; **(G1,G2)**: pork fat (cook for 20 min).

SEM results show that the sample surface of S1K2 is irregular, while the three-dimensional network structure became distinct in conjunction with an increase in the konjac content, as shown in Figure 2. The three-dimensional gel networks formed in the S1K4 were loose and rough with large, uneven internal pores. However, S1K6 still displayed significant gaps. With increased protein content, the 5% SPI network structures were denser while the mesh was smaller. However, S5K6 displayed dense lamellar structures instead of network structures, which could be attributed to the high solid content, preventing the sample matrix from forming a network structure. In Figures 2G1,G2 are the microstructure of pork fat cooked for 20 min. The collagen network structure is clearly visible, showing a surface covered in a thick layer of grease. According to the SEM photos, S1K4 is closest to pork fat because the pore size is closest to pork fat. The results showed that the micropore size of S1K4 network structure was similar to that of natural fat, resulting in similar sensory and texture characteristics.

**Figure 3:**
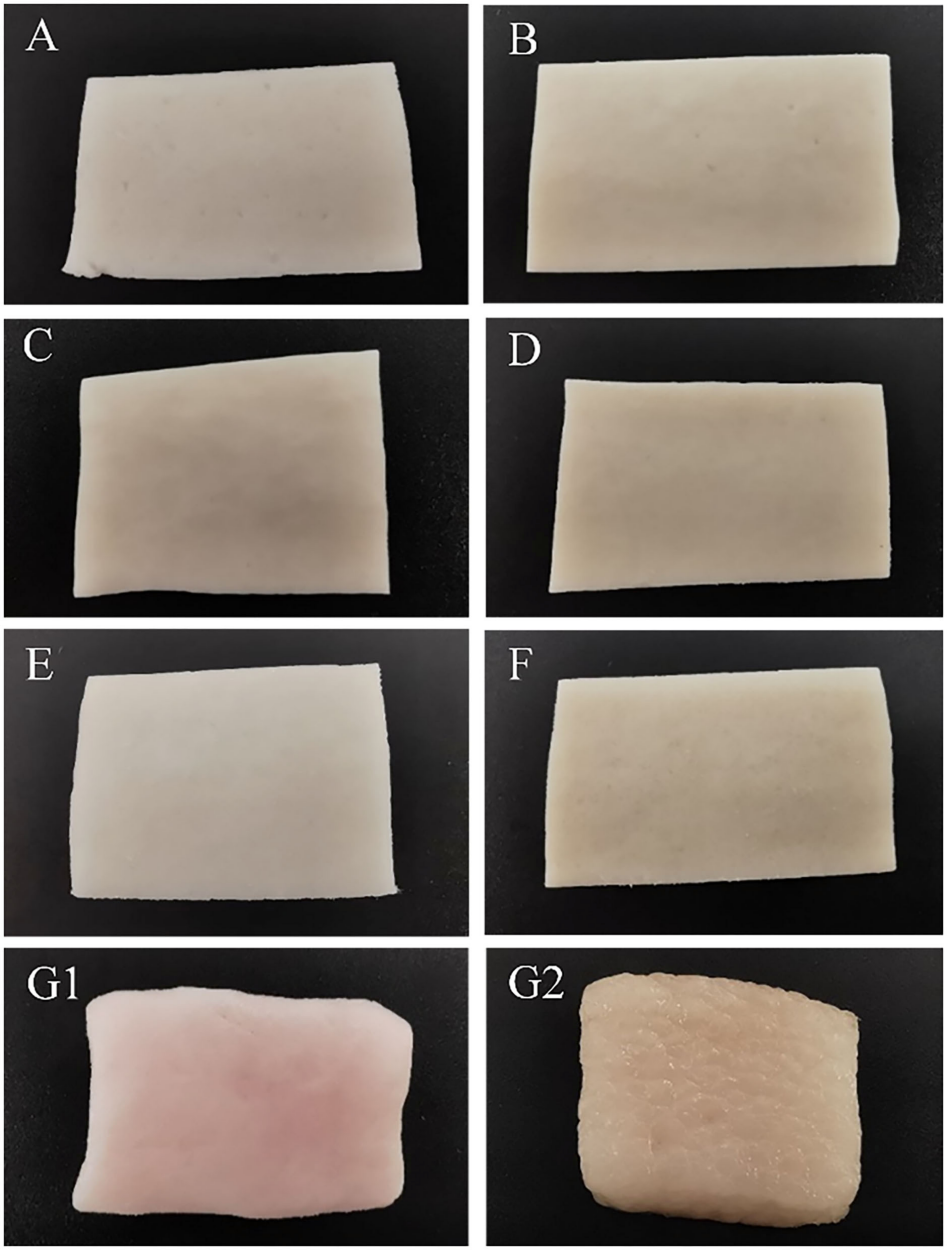
Photographic images of samples with different protein content and konjac content. **(A)** S1K2; **(B)** S5K2; **(C)** S2K4; **(D)** S5K4; **(E)** S1K6; **(F)** S5K6; **(G1)** pork fat; **(G2)** pork fat (cook for 20 min).

Figure 3 shows the appearance images of fat substitutes with different konjac and protein contents and pork fat cooked for 20 min. With the increase of protein content, brightness decreased, yellow increased, and red did not change significantly, which was consistent with the result of chromaticity.

### Sensory Evaluation

Table 2 shows the sensory evaluation results of samples with different konjac and protein contents. The results showed that S1K2 had the highest juice sense score compared with other fat substitutes, which may be due to the minimum content of konjac and protein and more water retained by konjac. With increased protein content and konjac content, the sense of juiciness decreased gradually, and the overall acceptability decreased. This is consistent with the result that the improvement of juiciness improved the overall acceptability of the final product in the study of fat-substitutes in Chinese Cantonese-style sausage (29). S5K4 and S5K6 scores are low, which is difficult for consumers to accept. There is no significant difference between S1K4 and S1K6, and the scores of chewiness and taste are high. Overall, S1K4 has the highest chewiness and acceptability scores.

**Table 2:** Sensory score results of the fat substitutes with different protein and konjac content levels and natural meat fat.

**Sample**	**Juiciness**	**Chewiness**	**Texture state**	**Overall acceptability**
S1K2	7.93 ± 0.96^a^	5.83 ± 2.01^bc^	5.13 ± 1.77^a^	5.93 ± 1.9^bc^
S1K4	6.07 ± 1.91^b^	7.57 ± 1.43^a^	6.67 ± 1.72^b^	7.33 ± 1.60^a^
S1K6	5.53 ± 1.88^b^	7.53 ± 1.41^a^	7.07 ± 1.28^b^	7.20 ± 1.52^a^
S5K2	5.27 ± 1.83^b^	7.07 ± 2.02^ab^	7.13 ± 1.51^b^	7.00 ± 1.36^ab^
S5K4	3.33 ± 1.45^c^	5.70 ± 1.94^bc^	7.00 ± 1.96^b^	5.33 ± 1.72^c^
S5K6	2.07 ± 1.39^d^	4.53 ± 2.53^c^	6.93 ± 2.76^b^	4.00 ± 1.46^d^
Natural fat (cook for 20 min)	4.97 ± 1.22^b^	7.33 ± 2.01^a^	7.60 ± 1.15^b^	6.60 ± 1.44^ab^

*Results are presented as mean values ± standard deviation (n = 15). Different lowercase letters indicate significant differences among different samples*.

### Texture Analysis

The mechanical properties of food are very important for consumers to perceive its texture characteristics. It can be seen from Table 3 that the texture data of most samples are similar to the pork fat cooked for 20 min, so it is possible to be used as potential fat substitute. With the increase of protein content, the hardness of the samples increased gradually, the cohesiveness decreased slightly, but the springiness did not change significantly. With the increase of konjac content, the springiness of fat substitutes with 1% SPI content increased significantly. The samples containing 5% SPI fat substitutes can better simulate the hardness of pork fat boiled for 20 min, and the cohesiveness of all samples is generally higher than that of pork fat. This may be due to the tight binding between protein and polysaccharide network matrix in fat substitutes, which has been reported in the emulsion stabilized by konjac glucomannan and SPI together (30). Under the same konjac content, the hardness of samples with high SPI content is higher, which is consistent with the study of Dreher et al. (31). That the hardness of samples with high SPI content under the same solid fat content is higher, because the number of proteins covalently crosslinked by TG is increased. The results show that protein/polysaccharide compound fat substitutes can change their mechanical properties by adjusting the amount of protein and konjac.

**Table 3:** The TPA of the fat substitutes with different protein and konjac content levels and natural meat fat.

**Sample**	**Hardness (g)**	**Cohesiveness (-)**	**Springiness (mm)**
S1K2	234.50 ± 7.05^a^	0.81 ± 0.02^c^	10.71 ± 0.11^a^
S5K2	1,485.00 ± 55.67^cd^	0.69 ± 0.05^b^	11.67 ± 0.45^a^
S1K4	689.40 ± 14.79^ab^	0.80 ± 0.04^c^	11.21 ± 0.45^a^
S5K4	1,589.00 ± 24.32^d^	0.69 ± 0.02^b^	11.23 ± 0.51^a^
S1K6	999.00 ± 137.89^bc^	0.78 ± 0.04^bc^	16.34 ± 5.09^b^
S5K6	2,984.50 ± 47.19^e^	0.54 ± 0.07^a^	10.98 ± 3.03^a^
Natural fat (cook for 20 min)	1,666.50 ± 142.13^d^	0.54 ± 0.16^a^	11.39 ± 0.42^a^

*Results are presented as mean values ± standard deviation (n = 4). Different lowercase letters indicate significant differences among different samples*.

### Analysis of Oral Tribology

**Figure 4:**
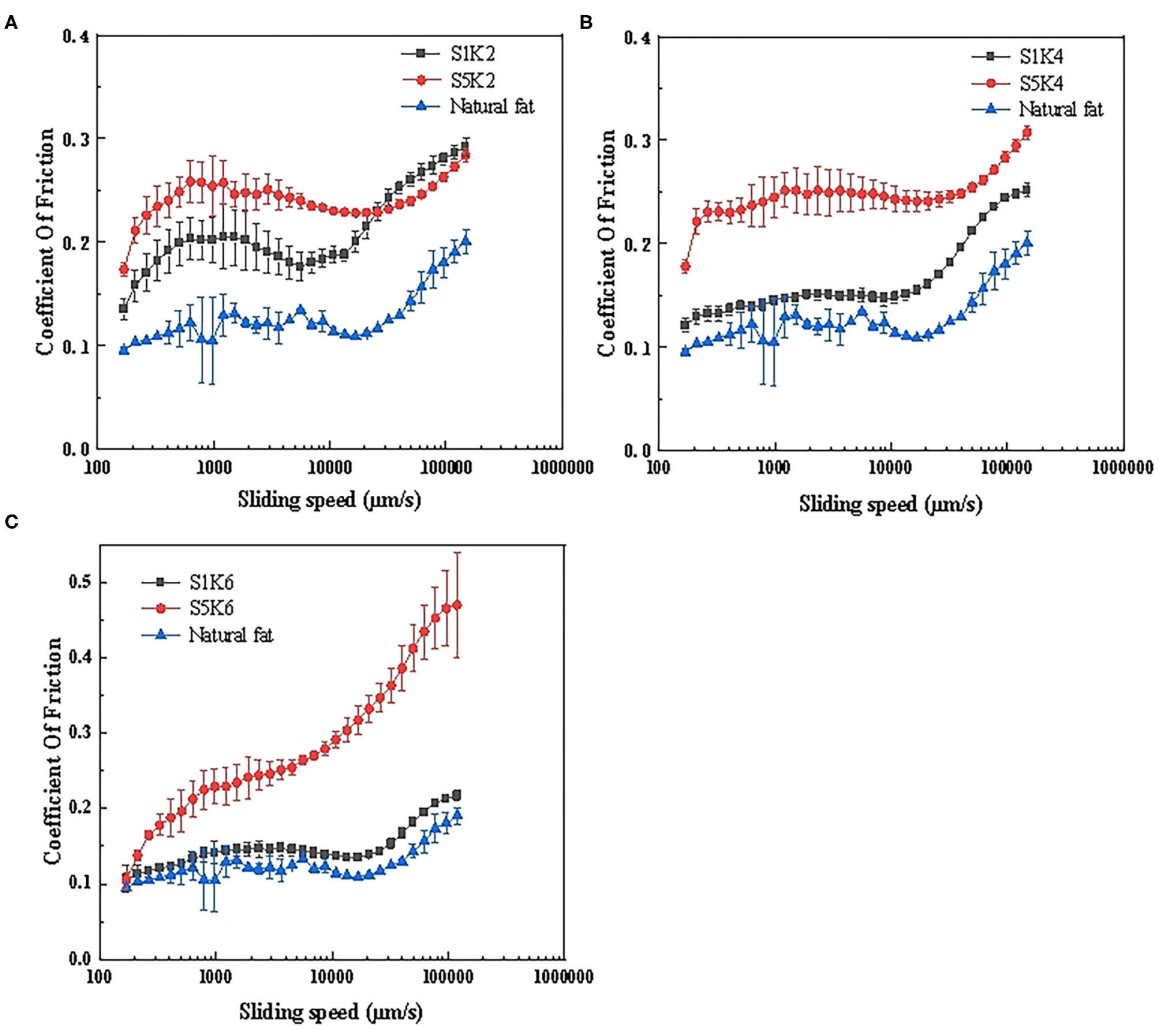
Friction curves of samples with different protein content and konjac content. **(A)** S1K2, S5K2, pork fat (cook for 20 min); **(B)** S1K4, S5K4, pork fat (cook for 20 min); **(C)** S1K6, S5K6, pork fat (cook for 20 min) (*n* = 3).

Tribology can be used to understand the lubrication performance of fat substitute and become a powerful screening tool. Understanding the friction behavior of protein substitute under frictional stress is essential for simulating the taste of natural meat fat. The friction curves of samples with different protein and konjac contents are shown in Figure 4. It can be seen that the overall friction curve trend of the samples is roughly the same as natural fat, and the curve trend of 1% SPI samples is closer to natural fat. Blue line shows the oral tribological results of cooking natural pork fat for 20 min. Since the natural pork fat contained a large amount of oil, it provided lubrication during the friction process, reducing the friction coefficient. The friction coefficient rose with an increase in the konjac content, causing the friction curve to move upward. Therefore, within this range, the content level of konjac increases and the friction coefficient increases, and the taste of the samples was rough (32). This could be attributed to the fact that higher konjac flour content increased the matrix viscosity, rendering the matrix more compact, thus increasing the friction coefficient. The friction coefficient of the 5% SPI was generally higher than the 1% SPI. This may be ascribed to the interaction between the increased protein content and other substrates, leading to the formation of aggregates in the closed space, possibly forming large particles that elevated the friction coefficient (33). The friction coefficient of 1% SPI samples is small, close to the pork fat, and the trend of friction curve is consistent with the pork fat. The friction coefficient of S1K6 is close to that of pork fat, which can better simulate fat, which is consistent with the sensory evaluation results.

### Color Analysis

Color is a primary factor affecting product purchases by consumers (34) and therefore, it is an essential parameter during the development of fat substitute and meat products. As shown in Table 4, higher protein content gradually decreased the brightness, causing the color to become darker. Although the redness value did not change significantly, the yellowness value displayed a substantial increase along with the change of protein content. Furthermore, a higher konjac content level caused a gradual decline in the brightness but had little effect on the redness and yellowness values. The yellowness value of the fat substitutes containing 5% SPI was similar to that of raw natural pork fat and fat boiled for 20 min.

**Table 4:** The color parameters of the fat substitutes with different protein and konjac content levels and natural meat fat.

**Sample**	**L**	**a**	**b**
S1K2	89.38 ± 0.10^a^	0.60 ± 0.09^c^	7.47 ± 0.10^a^
S5K2	81.66 ± 0.59^e^	0.57 ± 0.19^c^	14.07 ± 0.05^de^
S1K4	87.06 ± 0.29^b^	0.72 ± 0.06^c^	8.09 ± 0.14^b^
S5K4	81.77 ± 0.14^e^	0.69 ± 0.09^c^	13.64 ± 0.13^d^
S1K6	83.46 ± 0.21^d^	0.35 ± 0.05^b^	8.31 ± 0.09^b^
S5K6	78.09 ± 0.49^f^	0.73 ± 0.06^c^	14.56 ± 0.09^e^
Natural fat (cook for 20 min)	75.82 ± 0.24^g^	−0.47 ± 0.36^a^	15.12 ± 1.00^f^

*Results are presented as mean values ± standard deviation (n = 4). Different lowercase letters indicate significant differences among different samples*.

### DSC Analysis

**Figure 5:**
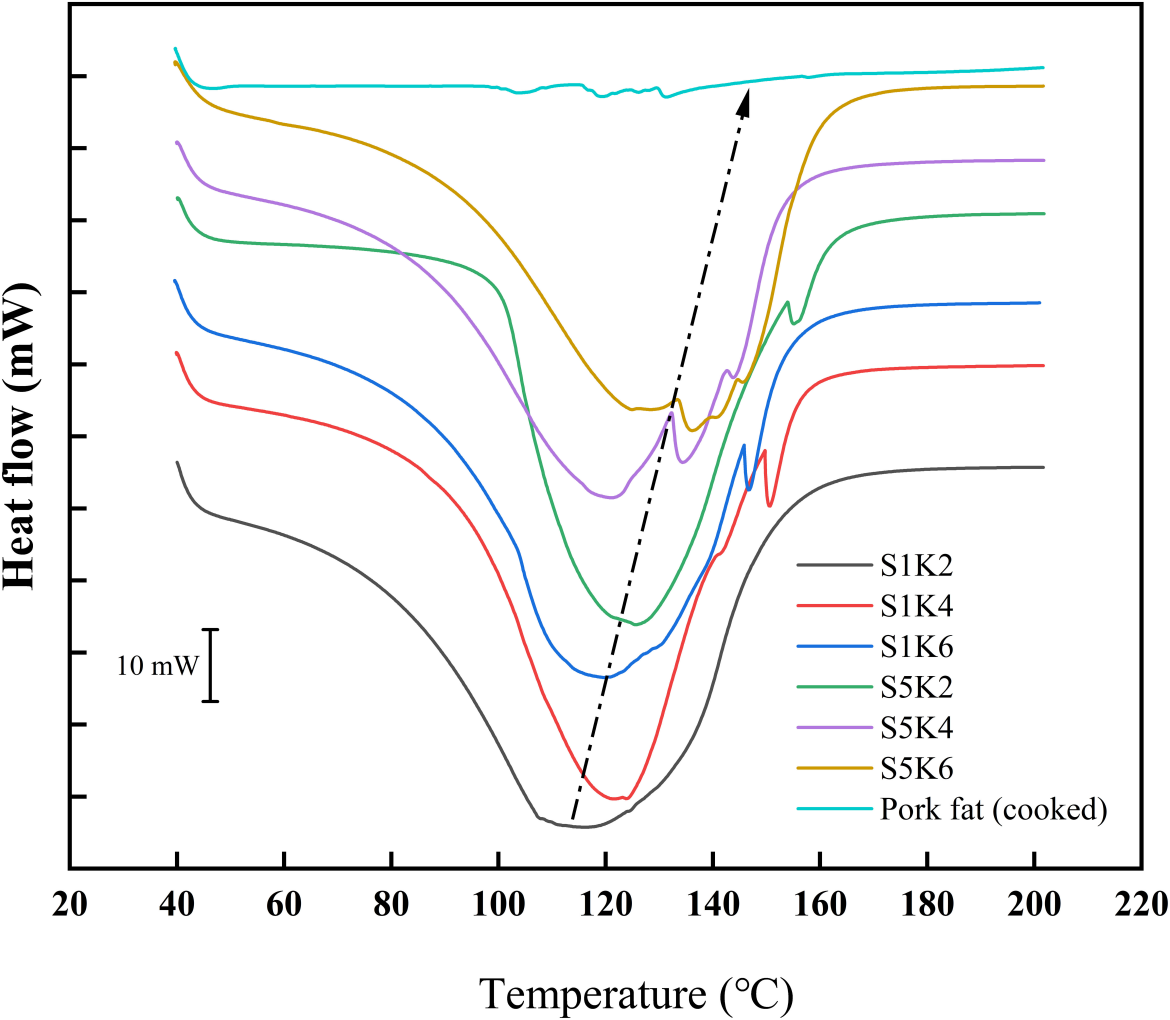
DSC curves of samples with different protein content and konjac content and pork fat (cook for 20 min).

The DSC curves of samples with different protein content and konjac content and the natural pork fat are shown in Figure 5. S1K2 has a peak at about 118°C. With the increase of konjac and SPI content, the peak value of fat substitutes gradually moves to a higher temperature. It may be that the increase of konjac and SPI content increases the adhesiveness of the samples and the crosslinking structure is closer, thus improving the thermal stability. The thermal trend of DSC curve of natural fat is quite different from that of fat substitute, which may be due to the great difference of water content between them. However, since the majority of the thermal peaks happened above 37°C, it will only impact the cooking behavior instead of the sensory attributes once cooked. The water content of pig adipose tissue is about 20% (35), while the water content of the sample is about 70%.

### Rheological Analysis

**Figure 6:**
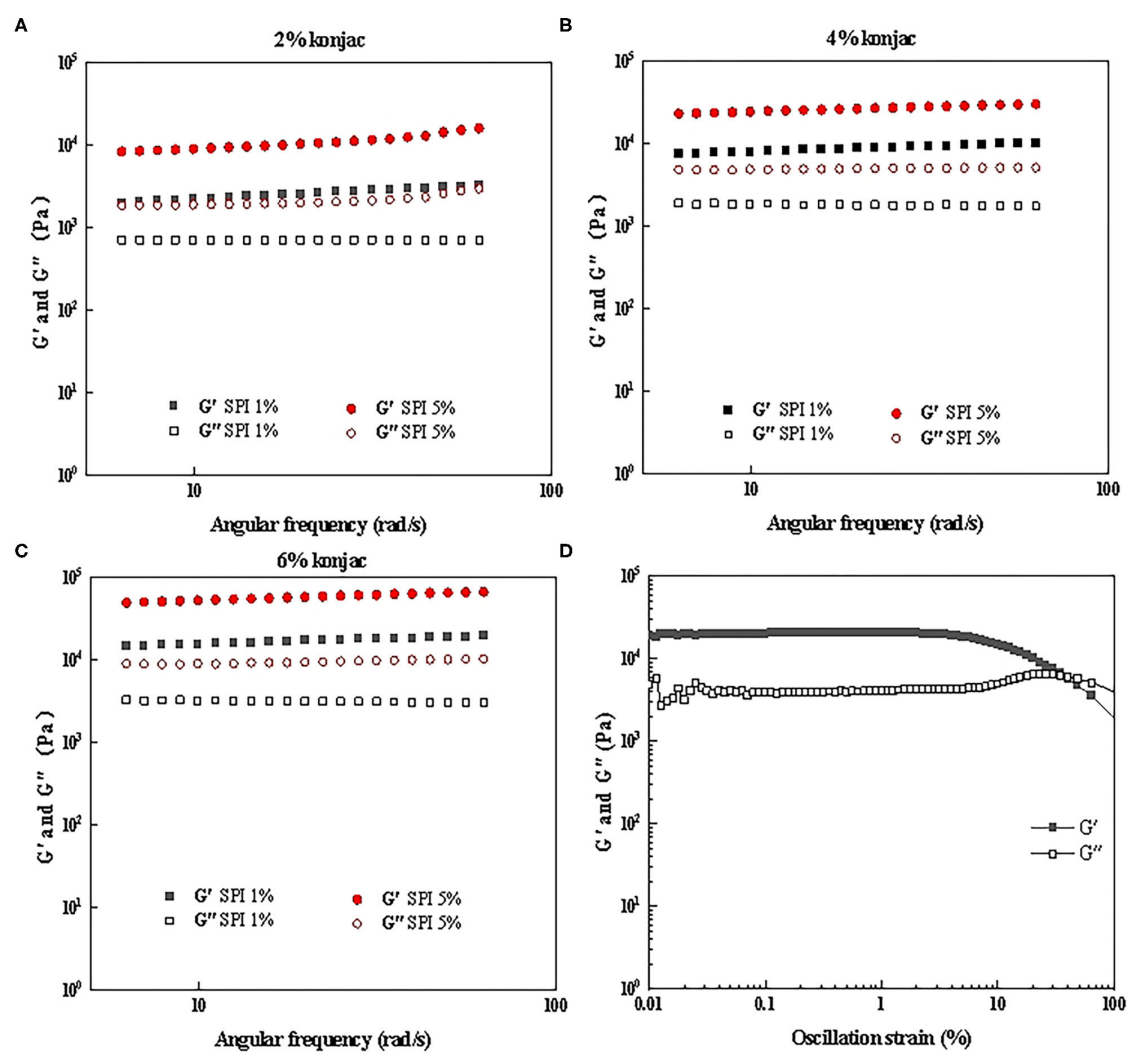
The frequency scanning results of samples with different protein content and konjac content. **(A)** S1K1, S5K1; **(B)** S1K4, S5K4; **(C)** S1K6, S5K6; **(D)** Amplitude scanning results of S5K4.

**Figure 7:**
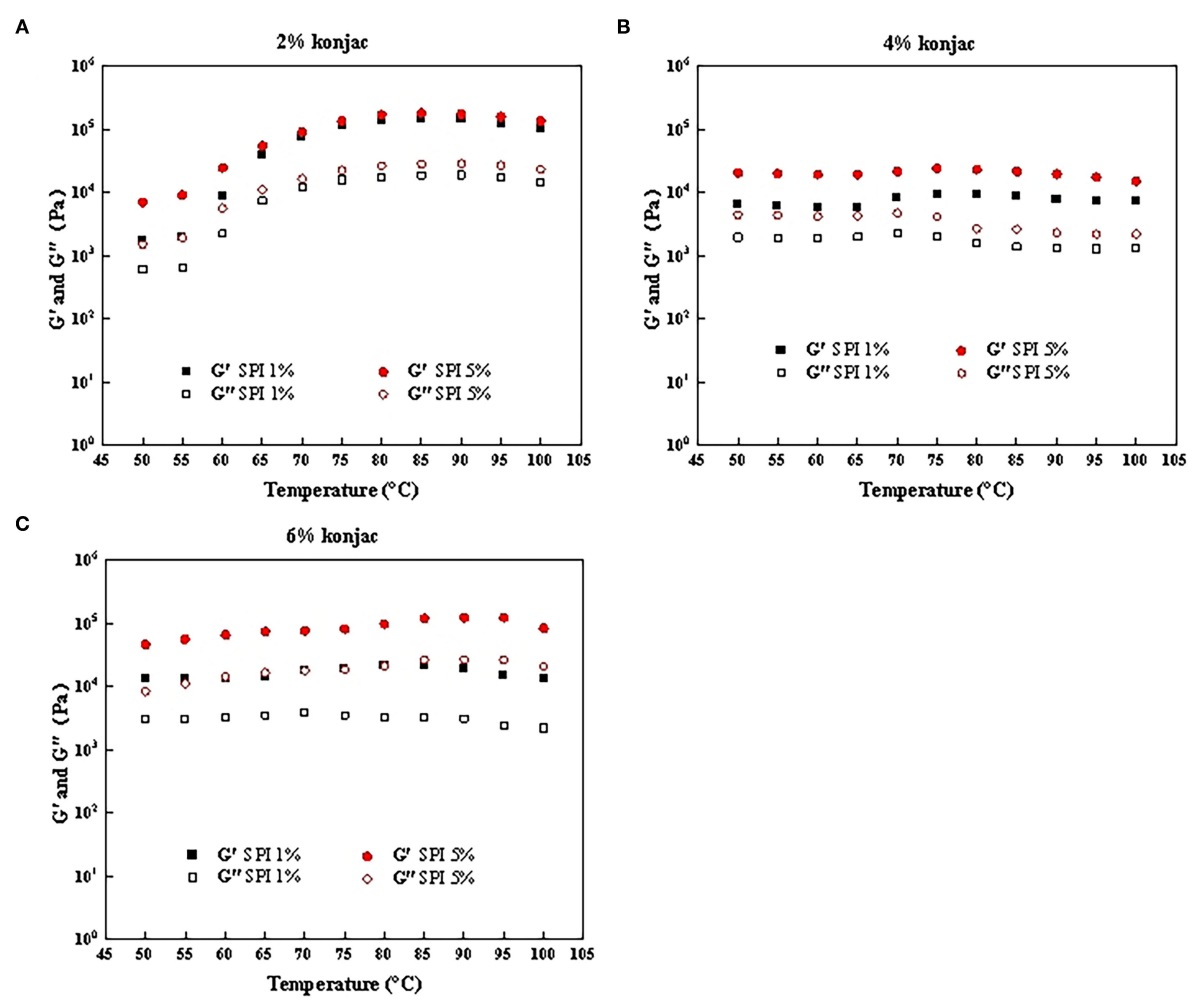
The temperature scanning results of samples with different protein content and konjac content. **(A)** S1K1, S5K1; **(B)** S1K4, S5K4; **(C)** S1K6, S5K6.

The impact of the protein and konjac content on the rheological properties of the SPI/polysaccharide composite gel was further investigated via amplitude scanning, frequency scanning, and temperature scanning. Figures 6A–C, 7A–C show the *G'* and *G”* as strain and temperature functions during frequency scanning and temperature scanning for different konjac content levels at 1% SPI and 5% SPI.

#### Frequency Scanning

The linear viscoelastic (LVE) region is characterized by the downward bending of the storage modulus *G'*. The measured *G'* and *G”* were functions of strain, as shown in Figure 6D, and the linear viscoelastic region was taken as below 1% strain in this study.

All samples showed gel-like characteristics (*G'* > *G”*) as shown in Figures 6A–C. For samples with different konjac content, the increase of protein content leads to the increase of *G'* and *G*″, and the increasing range of *G'* is greater than *G*″. This may be because the increase of protein content makes the network structure more closely bounds. Furthermore, higher konjac content also resulted in a substantial rise in the two moduli, which is consistent with the trends shown in previous study (36, 37). These rheological changes were consistent with the obvious changes of hardness in TPA (Table 3), which has been discussed in section 3.3. In addition, all the samples showed little dependence on the frequencies indicating no effect of the timescale over which the samples were probed. This result is consistent with the frequency scanning results of fat crystal network formed by transglutaminase induced crosslinking with SPI, rapeseed oil and hydrogenated rapeseed oil as raw materials in the study of simulated fat by Dreher et al. (2). However, it was slightly different from the low-fat pork liver Pâtés (a kind of Pie) enriched with n-3 PUFA/konjac gel studied by Delgado Pando et al. (38). This difference may be due to the difference in prescription, adding pork backfat and pork liver in their formula. It was also show that elastic modulus was greater than loss modulus (G' > G”), and the two moduli were highly dependent on the frequency.

#### Temperature Scanning

Comparing and analyzing the rheological properties of the fat substitutes help to better understand the influence of different protein and konjac concentrations on the sample structures during heat treatment. Similar to the frequency scanning results, the *G'* generally exceeded the *G*″, while both increased in conjunction with higher soybean protein and konjac content (Figures 7A–C).

The enzyme TG (glutaminyl-peptide γ-glutamyltransferase, EC 2.3.2.13) catalyzes the acyl transfer reaction between protein-bound glutaminyl residues and primary amines (39). The *G'* of the samples containing 2% konjac began to increase at around 65°C, probably due to the network structure induced by the covalent TG enzyme cross-linking of SPI, changing the weak network of the paste to a more ordered gel matrix. The overall change trend of G' and G” of 2% konjac and 6% konjac samples was the same. It shows no obvious change at 50–60°C, and the main rheological change occurs above 60°C. This result was consistent with those obtained by Jimenez-Colmenero et al. in which the main rheological changes caused by konjac content were evident above 60°C with a slight increase in the *G'* (3). The G' of the samples displayed a distinct increase about 60°C, reaching the maximum value at 95°C, indicating the formation of a rigid elastic matrix of heat-induced protein aggregation/gels, which is consistent with the results of Delgado-Pando et al. (38). However, when the temperature exceeds 95°C, G' and G” tend to decline. It may be that the temperature was too high, the network was unstable and the hydrogen bond was destroyed (40).

## Conclusion

This study shows that SPI/polysaccharide composite gel can be used to simulate three-dimensional block fat. Furthermore, the SPI and konjac significantly affect the rheological behavior of the protein/polysaccharide composite gel, as well as the structure of protein/polysaccharide compound fat substitute and oral tribological analysis. A higher konjac content level significantly increases the hardness and friction coefficient of the fat substitute. Appropriate addition can simulate the texture and greasy taste of natural fat. Although the appearance of the fat substitute is similar to that of natural fat, it lacks redness. For the first time, an oral friction analysis of the SPI/polysaccharide emulsion fat substitute is conducted to compare its textural with that of natural pork fat. In addition, research involving the acceptability of sensory evaluation will be the key to the successful implementation of this new product. Further research should focus on the cooking characteristics of protein/polysaccharide composite fat substitutes, such as water holding capacity, cooking loss, and appearance while encapsulating natural pigments to increase the redness value, shelf-life, and nutritional characteristics of protein/polysaccharide composite fat substitutes.

## Data Availability Statement

The original contributions presented in the study are included in the article/supplementary material, further inquiries can be directed to the corresponding author/s.

## Author Contributions

LH designed the research and carried out pre experiments. YR and QZ tested and analyzed physical and chemical properties of fat substitutes. LH and JC analyzed the data. LH and HL wrote the initial manuscript, with contributions from YW and XL. All authors contributed to the article and approved the submitted version.

## Funding

The National Key Research and Development Program of China, Grant/Award Number: 2021YFC2101400.

## Conflict of Interest

JC is employed by Plant Meat (Hangzhou) Health Technology Limited Company. The remaining authors declare that the research was conducted in the absence of any commercial or financial relationships that could be construed as a potential conflict of interest.

## Publisher's Note

All claims expressed in this article are solely those of the authors and do not necessarily represent those of their affiliated organizations, or those of the publisher, the editors and the reviewers. Any product that may be evaluated in this article, or claim that may be made by its manufacturer, is not guaranteed or endorsed by the publisher.
